# Helminthofauna of *Batrachoides surinamensis* (Batrachoidiformes: Batrachoididae) from estuaries of the Amazon in Pará, Brazil

**DOI:** 10.1590/S1984-29612025064

**Published:** 2025-11-17

**Authors:** Adriene Martins da Silva, Ricardo Luis Sousa Santana, Elaine Lopes de Carvalho, Elane Guerreiro Giese

**Affiliations:** 1 Universidade Federal Rural da Amazônia – UFRA, Instituto da Saúde e Produção Animal – ISPA, Laboratório de Histologia e Embriologia Animal, Belém, PA, Brasil

**Keywords:** Helminths, Batrachoididae, gastrointestinal helminth, Brazilian Amazon, peixe, Batrachoididae, helmintos gastrointestinais, Amazônia brasileira

## Abstract

This research aimed to identify the species that make up the helminth fauna of *Batrachoides surinamensis*, a commercially important fish on Marajó Island and the Bragança region, Pará, Brazil. A total of 146 specimens of *B. surinamensis* from Marajó Island and 60 specimens from Bragança were analyzed between 2021 to 2024. The samples were acquired from artisanal fishers at the time of landing. The helminth parasites found were processed for analysis by light and scanning electron microscopy. Taxonomic identification was performed using phylum keys, and scientific articles with original descriptions and redescriptions of species were used to identify the taxa present. Helminths were quantified to determine prevalence, mean intensity of infection and mean abundance parameters. In the collections from Marajó Island, of Nematoda had the highest prevalence at 76.71%, followed by Trematoda at 25.34%, Cestoda at 86.98% and Acanthocephala at 0.68%. In the collection from Bragança of Nematoda had the highest prevalence at 83.33%, followed by Cestoda at 40%, Trematoda at 81.66% and no Acanthocephala were recorded. It was possible to make the first record of the gastrointestinal helminths of *B. surinamensis* from Marajó Island and the Bragança region in the state of Pará.

## RESUMO

Esta pesquisa teve como objetivo identificar as espécies que compõem a helmintofauna de *Batrachoides surinamensis*, um peixe de importância comercial na Ilha de Marajó e região de Bragança, Pará, Brasil. Um total de 146 espécimes de *B. surinamensis* da Ilha de Marajó e 60 espécimes de Bragança foram analisados ​​durante o período de 2021 a 2024. As amostras foram adquiridas de pescadores artesanais no momento do desembarque. Os helmintos parasitos encontrados foram processados para análise por microscopias de luz e eletrônica de varredura. A identificação taxonômica foi realizada usando chaves de acordo com o Filo, e artigos científicos com descrições originais e redescrições de espécies para identificar os táxons presentes. Os helmintos foram quantificados para determinar os parâmetros de prevalência, intensidade média de infecção e abundância média. Houve maior prevalência de Nematoda com 76,71%, seguido de Trematoda com 25,34%, Cestoda 86,98% e Acanthocephala 0,68%, nas coletas da Ilha de Marajó. E na coleta de Bragança houve maior prevalência de Nematoda com 83,33%, Cestoda com 40%, Trematoda 81,66% e Acanthocephala não foram registrados. Foi possível fazer o primeiro registro de helmintos gastrintestinal de *B. surinamensis* da Ilha de Marajó e região de Bragança no estado do Pará.

## Introduction

Brazil has a rich helminth fauna in fish that is already known and is considered one of the hotspots of parasite biodiversity in South America (Eiras et al., 2010; Luque et al., 2017). Studies on fish parasites are of great importance, not only for understanding their helminth fauna and for functioning as excellent indicators of biodiversity (Sures et al., 2017), but also for their health, economic, and social importance, since many of these fish are used for fisheries production, commercialization, and consumption by local populations (Amato et al., 1990; Eiras, 1994; Garcia et al., 2017; Monteiro et al., 2019).

The fish family Batrachoididae in Brazil is represented by 6 genera and 13 species (Menezes et al., 2003). These include the Pacuma Toadfish *Batrachoides surinamensis* (Bloch & Schneider 1801), which is normally found in shallow areas with brackish waters of estuarine environments (Léopold, 2004). Garcia et al. (2017) provided the first record of the species on the northeastern coast of Brazil, in the state of Rio Grande do Norte. The species *B. surinamensis* is popularly known pacamão, pacamum or peixe-sapo.

The solitary behavior of this species, combined with its benthic lifestyle, slow movements, cryptic coloration, and inconspicuous habits, is strongly related to its foraging strategy based on ambushing prey (Piorski & Nunes, 2010). It is a demersal estuarine species, commonly found in sandy and muddy bottoms of shallow, warm waters. Although predominantly solitary, the species forms pairs during the reproductive period and intensifies its feeding activity at night (Espírito Santo et al., 2005; Melo et al., 2015; Mrceniuk et al., 2021).

Knowledge of endoparasite fauna is still scarce in Brazil, with the species *Hysterothylacium fortalezae* (Klein, 1973) (= *Contracaecum fortalezae*) having been found parasitizing *Scomberomorus brasiliensis* Collete, Russo & Zavala-Camim, 1978 (Klein, 1973) and *Hysterothylacium reliquens* (Norris & Overstreet, 1975) in *B. surinamensis* Bloch & Schneider, 1801 (Deardorff & Overstreet, 1980) also having been recorded so far. Eiras et al. (2017) recorded *H. reliquens* (Norris & Overstreet, 1975) in *Batrachoides* spp. located in the stomach and intestine of these fish in Brazil, Colombia and Guyana.

Different species of fish helminths can lead to pathological changes, resulting in high mortality rates, reduced catches or commercial losses, and the losses can be incalculable (Eiras, 1994). Furthermore, some parasites are of great importance in the hygienic-sanitary inspection of fish to be sold (Amato et al., 1990). Although the toadfish is a fish sold in street markets in the Northeast (Garcia et al., 2017) and is consumed by the riverside population of the Brazilian Amazon (Monteiro et al., 2019). Despite limited previous records of specific helminths in *B. surinamensis*, a comprehensive survey of its helminth fauna in the Amazon region is lacking.

This research seeks to identify the species that make up the helminth fauna of *B. surinamensis*, a fish of commercial importance on Marajó Island and in the Bragança region, Pará, Brazil.

## Material and Methods

### Study area

Marajó Island is the largest island in the archipelago at the mouth of the Amazon River, with an area of ​​50,000 km^2^, located in the northeastern portion of the State of Pará. It is bathed by the muddy waters of the Amazon River, Marajó Bay and the Tocantins River, as well as the Atlantic Ocean, and is one of the most important estuaries in Brazil (OTCA, 2018). The municipality of Soure has an area of ​​2,857 km^2^, and the population is estimated at 25,218 people (Brasil, 2024). The Soure Marine Extractive Reserve (RESEXMAR-Soure) has an area of ​​approximately 27 thousand hectares distributed in two discontinuous areas: the Soure Mangrove (the largest) and the Saco River Mangrove (Neves, 2020).

Bragança is one of the oldest municipalities in the state of Pará and is located on the left bank of the Caeté River. The municipality has a land area of 2,124.734 km^2^ and a population of 123,082 hab. It is characterized by a humid equatorial zonal climate. (FAPESPA, 2023).

**Figure 1:**
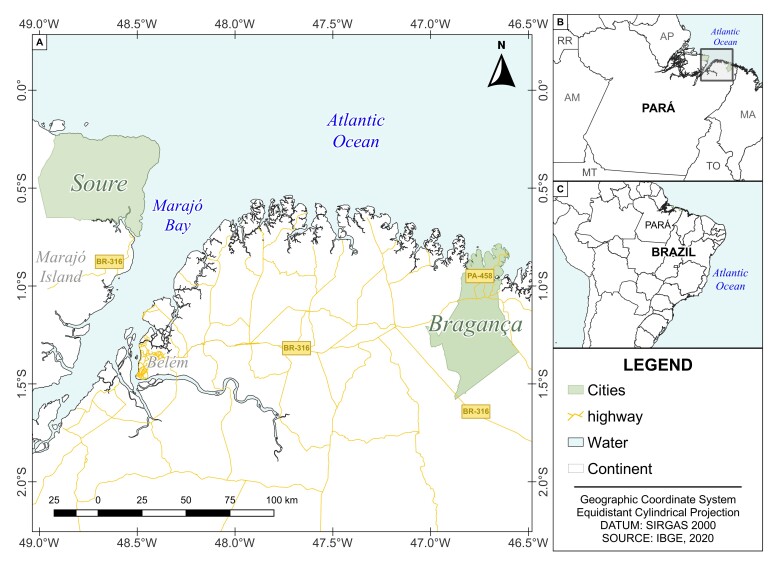
Amazon Biome, highlighting (green) the municipalities of Soure and Bragança.

*B. surinamensis* specimens were obtained between June 2021 to August 2024. A total of 206 specimens (adult) were acquired from artisanal fishers at the time of landing. The fish came from the municipalities of Soure on the island of Marajó, and from the municipality of Bragança, both in the state of Pará (Figure 1).

### Parasitological examination

The fish were individually placed in plastic bags, packaged and kept refrigerated in isothermal boxes with ice and sent to the Animal Histology and Embryology Laboratory (LHEA) of the Institute of Animal Health and Production (ISPA) at UFRA, for 4 hours of travel from Soure to Belém, and for 3.5 hours from Bragança to Belém. In the laboratory, the animals were cataloged and stored in a refrigerator. To obtain data regarding parasitic indices, three body regions of the samples obtained were analyzed: body musculature (muscle mass on each side of the spine), abdominal musculature (ventral musculature lining the abdominal cavity) and parietal abdominal serosa according to Molento et al. (2017).

In the laboratory, the fish were necropsied, starting with a longitudinal incision from the cloacal opening to the mouth, exposing the coelomic cavity and organs. In addition to the musculature, the tegument, mouth, gills, esophagus, stomach, liver, gallbladder, swim bladder, pyloric cecum, gonads, intestine, and cloaca were analyzed. After this, these organs were removed and individually placed in Petri dishes containing a 0.9% saline solution and analyzed using a Leica ES2 stereomicroscope (Wetzlar, Germany) to investigate the presence of parasites. All data were recorded on a necropsy form. The helminths were processed for analysis by light microscopy and scanning electron microscopy.

### Light microscopy

Specimens of the phyla Nematoda and Acanthocephala were collected, fixed in A.F.A solution (2% glacial acetic acid, 3% 37% formaldehyde and 95% 70% ethyl alcohol), clarified with Aman's Lactophenol or in glycerinated alcohol, and observed under a light microscope Leica DM 2500 (Wetzlar, Germany) between slide and a coverslip. Trematodes and cestodes were compressed between a slide and a coverslip in a Petri dish to which A.F.A. fixative was added. The compression time was assessed based on the thickness of the helminth (Oyarzún-Ruiz & González-Acuña, 2020). Live cestodes were placed in distilled water and refrigerated to die with their muscles relaxed (Amato & Amato, 2010).

The collected specimens of Digenea and Cestoda were stained with Gomori's Trichrome or Acetic Carmine, subjected to a regressive alcohol process, differentiated in salicylate, and mounted on permanent slides with Entellan^®^. The specimens of the Phylum Nematoda were subjected to dehydration in an ethanol series, clarified with lactophenol and placed under the microscope Leica DM 2500 (Wetzlar, Germany), between slide and coverslip for analysis according to Amato et al. (1991).

Subsequently, the helminths were observed under a microscope Leica DM 2500 (Wetzlar, Germany) with an attached digital capture system Leica ICC50 HD (Wetzlar, Germany) and measured under a microscope Leica DM 2500 (Wetzlar, Germany) with an attached clear camera, from which photomicrographs and morphological drawings were obtained, respectively. For this, 5×, 10× and 40× objectives were used, and if necessary, 100×.

### Scanning electron microscopy (SEM)

For SEM, parasites were fixed in 10% formaldehyde, washed in 0.2M phosphate buffer solution. Each one was washed for one hour, then post-fixed in 1% Osmium Tetroxide, dehydrated in progressive alcohol for one hour each (50%, 70%, 80%, 90%, 100%), and dried at the critical point of CO_2_, metallized with palladium-gold and observed in a TESCAN scanning electron microscope model VEGA 3 (Brno, Czech Republic) as per Carvalho et al. (2025).

### Taxonomic identification

To identify the helminths found, the following keys were used according to the Phylum: Nematoda according to Anderson et al. (2009); Platyhelminthes of the class Gibson et al. (2002) and Bray et al. (2008) The cestodes were determined according to Khalil et al. (1994); the acanthocephalans according to Petrochenko (1971), all with the help of publications in scientific journals.

### Statistical analysis

To determine the parasitism rates, these helminths were analyzed by prevalence (%), mean infection intensity (M_I_) and mean abundance (M_A_), according to Bush et al. (1997).

## Results

During the period from June 2021 to August 2024, 146 specimens of *B. surinamensis* were analyzed, from the municipality of Soure, Marajó Island, Pará. Of the 146 samples analyzed, 41.10% (n=60) belonged to female animals and 58.90% (n=86) to males. Of these specimens analyzed, parasitism was found in 92.46% (n = 135), and no parasitism was observed in 7.54% (n = 11). The Phylum Nematoda was the most representative group occurring in 76.71% (n = 112) of the fish, followed by Platyhelminthes – Class Trematoda with 25.34% (n = 37), Cestoda 86.98% (n = 127) and Acanthocephala 0.68% (n = 1).

During the period from June 2021 to July 2024, 60 specimens of *B. surinamensis* from the municipality of Bragança, Pará, were analyzed. Of the 60 samples analyzed, 48.33% (n = 29) belonged to female animals and 51.67% (n = 31) to males. Of the 60 specimens analyzed, parasitism was evidenced in 88.33% (n = 53). The Phylum Nematoda was the most representative group, occurring in 83.33% (n = 50) of the samples. Platyhelminthes – Cestoda Class came next with 40% (n = 24), Trematoda 81.66% (n = 49), and Acanthocephala were not recorded.

**Figure 2:**
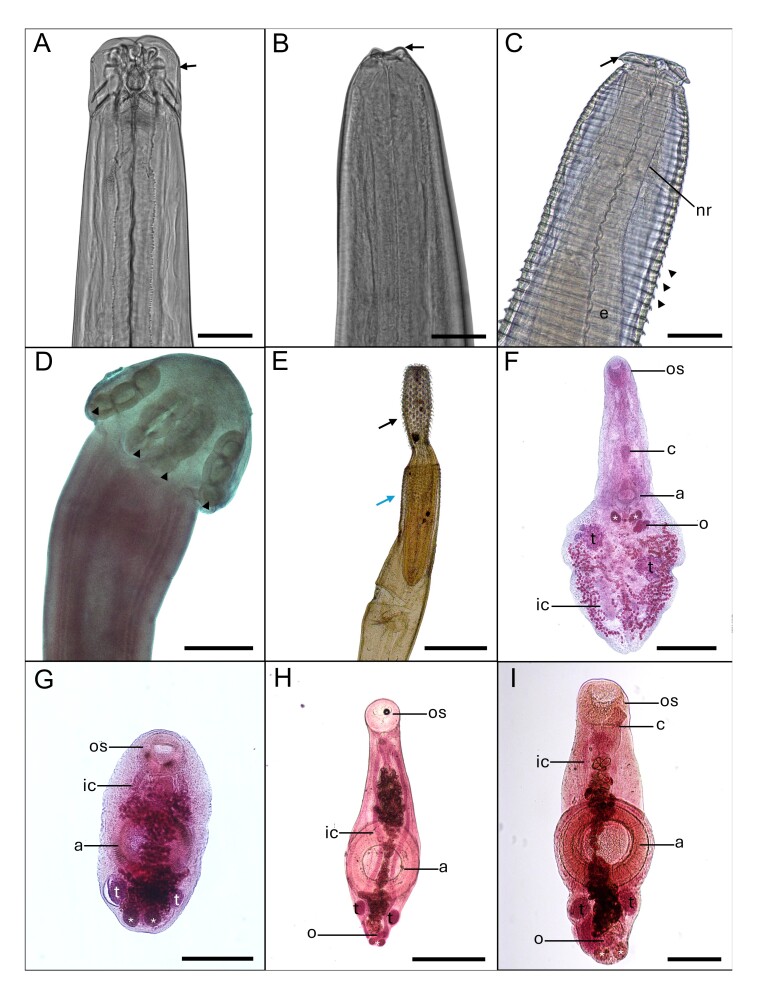
Helminths in *Batrachoides surinamensis*. A. *Hysterotylacium* sp. adult, three developed lips (arrow). Scale bar: 200 μm; B. *Hysterotylacium* sp. larval stage L4, with formation of lips (arrow). Scale bar: 50 μm; C. *Goezia* sp., lips (arrow), nerve ring (nr), esophagus (e) and cuticular spines on the striations of the body (arrowhead). Scale bar: 100 μm; D. *Gibsoniela* sp., trilocular suckers (arrowhead). Scale bar: 500 μm; E. *Serrasentis* sp., proboscis with hooks (black arrow) and collar of spines (blue arrow). Scale bar: 1 mm; F. *Phyllodistomum* sp., oral sucker (os), acetabulum (a), intestinal ceca (ic), vitellarium (*), testes (t) and ovary (o) (t). Scale bar: 500 μm. Staining: Gomory trichrome. G. *Gonocercella* sp. oral sucker (os), intestinal ceca (ic), acetabulum (a), vitellarium (*) and testes (t). Scale bar: 500 μm. Staining: Alcoholic carmine; H. *Derogenoides* sp. morphotype 1, oral sucker (os), intestinal ceca (ic), acetabulum (a), testes (t) and ovary (o). Scale bar: 500 μm. Staining: Alcoholic carmine; I. *Derogenoides* sp. morphotype 2, oral sucker (os), intestinal ceca (ic), acetabulum (a), testes (t) and ovary (o). Scale bar: 200 μm. Staining: Alcoholic carmine.

**Figure 3:**
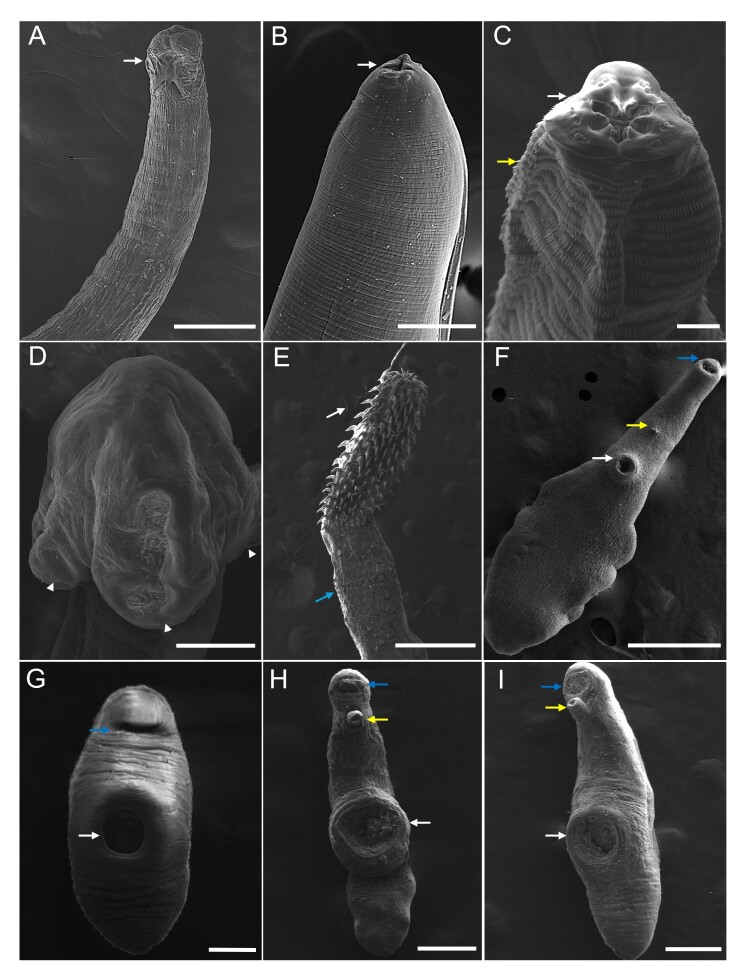
Scanning electron microscopy of *Batrachoides surinamensis* helminths. A. *Hysterotylacium* sp. adult, three developed lips (arrow). Scale bar: 200 μm; B. *Hysterotylacium* sp. larval stage L4, with formation of lips (arrow). Scale bar: 50 μm; C. *Goezia* sp., lips (arrow) and cuticular spines on the striations of the body (yellow arrow). Scale bar: 20 μm; D. *Gibsoniela* sp., trilocular suckers (arrowhead). Scale bar: 200 μm; E. *Serrasentis* sp., proboscis with hooks (arrow) and collar of spines (blue arrow). Scale bar: 500 μm; F. *Phyllodistomum* sp., oral sucker (blue arrow), acetabulum (arrow) and genital pore (yellow arrow). Scale bar: 500 μm. G. *Gonocercella* sp. oral sucker (blue arrow) and acetabulum (arrow). Scale bar: 500 μm; H. *Derogenoides* sp. morphotype 1, oral sucker (blue arrow), acetabulum (arrow) and genital pore with exposed cirrus (yellow arrow). Scale bar: 500 μm; I. *Derogenoides* sp. morphotype 2, oral sucker (blue arrow), acetabulum (arrow), and genital pore with exposed cirrus (yellow arrow). Scale bar: 500 μm.

In the two municipalities studied, there was no parasite infection in the fish muscles. The helminths recorded at the genus or species level are shown in Table 1 and represented in Figure 2. The basic morphometric data used for the identification of the helminths is in Table 2, and the SEM images are in Figure 3.

**Table 1:** Parasitism index in *Batrachoides surinamensis* from the municipality of Soure, Marajó Pará (n=146) and in the municipality of Bragança, Pará (n=60).

**Taxon**	**Site of infection**	**Host number**	**Number of parasites**	**Location**	**Prevalence (%)**	**Average Intensity of Infection**	**Average abundance**
**Phylum Nematoda**							
**Family Raphidascarididae**							
*Hysterothylacium* sp.	Intestine	20	104	S	13.6	5.2	0.7
		20	865	B	33.3	43.2	14.4
*Goezia* sp.	Stomach	3	15	S	2.0	5.0	0.1
**Family Anisakidae**							
*Anisakis* sp.	Intestine	20	314	S	13.6	15.7	2.1
	Liver	20	150	S	13.6	7.5	1.0
		10	103	B	16.7	10.3	1.7
**Phylum Platyhelminthes**							
**Class Trematoda**							
**Family Derogenidae Nicoll, 1910**							
*Derogenoides* sp. Type 1	Intestine	20	108	S	13.6	5.4	0.7
*Derogenoides* sp. Type 2	Stomach	10	147	S	6.8	14.7	1.0
*Gonocercella*	Stomach	12	25	S	8.22	2.08	0.17
**Family Gorgoderidae Looss, 1901**							
*Phyllodistomum* sp.	Cecum	2	38	S	1.4	19.0	0.2
		1	20	B	1.7	20.0	0.3
**Phylum Platyhelminthes**							
**Class Cestoda**							
**Family Protocepahilidae Mola,1929**							
*Gibsoniela* sp.	Intestine	50	1.317	S	34.0	26.3	8.9
		34	757	B	56.7	22.3	12.6
**Phylum Acanthocephala**							
**Family Isthmosacanthidae Smales, 2012**							
*Serrasentis* sp.	Stomach	1	5	S	3.42	5	0.03

S: Soure; B: Bragança.

**Table 2:** General characteristics of the genera of helminths Parasites of *Batrachoides surinamensis* in the Municipality of Soure and Bragança, State of Pará, Brazil.

**Caracteres**	***Hysterotylacyum* Ward & Magath, 1917**	***Hysterotylacyum* L3**	***Anisakis* Dujardin, 1845**	***Goezia* Zeder, 1800**
				
Body Length	48.48 (42.28 – 56.94) 3.34	15.53 (14.06 – 16.34) 0.42	11.54 (10.89 – 13.06) 0.41	4.56 (3.70 – 5.93) 0.42
Width	0.53 (0.46 – 0.56) 0.02	0.23 (0.17 – 0.28) 0.02	0.18 (0.14 – 0.23) 0.01	0.24 (0.02 – 0.32) 0.05
Nervous ring	0.85^a^ (0.73 - 1) 0.06	0.48^a^(0.40 – 0.57) 0.03	0.20^a^ (0.19 – 0.21) 0.004	0.21^a^ (0.19 – 0.23) 0.004
Esophagus	6.83 (5.94 – 8.03) 0.37	1.55 (1.21 – 1.71) 0.09	0.88 (0.79 – 0.91) 0.02	0.65 (0.60 – 0.71) 0.02
VL	0.29 (0.23 – 0.25) 0.02	0.09 (0.05 – 0.12) 0.01	0.34 (0.29 – 0.45) 0.03	0.08 (0.08 – 0.09) 0.004
VW	0.33 (0.31 – 0.35) 0.01	0.09 (0.07 – 0.11) 0.004	0.13 (0.09 – 0.19) 0.02	-
VA	1.94 (1.72 – 2.14) 0.08	8.03 (1.87 – 23.43) 3.90	-	-
Tail	0.18 (0.06 – 0.23) 0.03	0.23 (0.17 – 0.42) 0.05	0.08 (0.07 – 0.10) 0.004	0.10 (0.05 – 0.13) 0.01
Mucron	-	-	Absent	-
Thorns	-	-	-	Present
**Caracteres**	***Derogenoides* Type 1**	***Derogenoides* Type 2**	***Gonocercella* Manter, 1940**	***Phyllodistomum* Braun, 1899**
				
Body Length	1.85 (1.56 – 2.22) 0.12	1.53 (1.35 – 1.60) 0.04	1.28 (0.97 – 1.59) 0.10	1.53 (1.20 - 2) 0.13
OS ^(L)^	0.27 (0.25 – 0.31) 0.01	0.25 (0.22 – 0.28) 0.01	0.27 (0.20 – 0.33) 0.02	0.19 (0.17 – 0.21) 0.01
OS ^(W)^	0.27 (0.25 – 0.29) 0.01	0.23 (0.21 – 0.25) 0.004	0.21 (0.17 – 0.34) 0.03	0.15 (0.15 – 0.16) 0.004
A^(L)^	0.50 (0.45 – 0.55) 0.02	0.66 (0.40 – 1.05) 0.16	0.37 (0.24 – 0.63) 0.07	0.17 (0.12 – 0.21) 0.01
A^(W)^	0.46 (0.42 – 0.54) 0.02	0.58 (0.35 – 0.90) 0.13	0.31 (0.21 – 0.61) 0.08	0.13 (0.12 – 0.15) 0.004
AT ^(L)^	0.17 (0.14 – 0.19) 0.004	0.14 (0.13 – 0.15) 0.004	0.13 (0.12 – 0.15) 0.004	0.21 (0.13 – 0.24) 0.02
AT ^(W)^	0.15 (0.13 – 0.16) 0.01	0.11 (0.07 – 0.14) 0.01	0.11 (0.10 – 0.12) 0.004	0.24 (0.21 – 0.26) 0.01
PT ^(L)^	0.17 (0.15 – 0.18) 0.004	0.17 (0.16 – 0.17) 0	0.19 (0.16 – 0.22) 0.01	0.25 (0.20 – 0.27) 0.01
PT ^(W)^	0.13 (0.09 – 0.15) 0.01	0.13 (0.08 – 0.14) 0.01	0.16 (0.13 – 0.19) 0.01	0.23 (0.16 – 0.26) 0.004
Pharynx^(L)^	0.12 (0.09 – 0.15) 0.01	0.09 (0.07 – 0.10) 0.004	0.12 (0.09 – 0.17) 0.01	0.18 (0.15 – 0.20) 0.01
RVt ^(L)^	0.12 (0.07 – 0.14) 0.01	0.08 (0.07 – 0.10) 0.004	0.12 (0.10 – 0.15) 0.004	0.13 (0.11 – 0.15) 0.01
LVt ^(L)^	0.13 (0.08 – 0.15) 0.01	0.08 (0.07 – 0.09) 0.004	0.11 (0.10 – 0.12) 0.01	0.14 (0.1 – 0.17) 0.01
Ovary^(L)^	0.14 (0.07 – 0.19) 0.02	0.24 (0.08 – 0.85) 0.004	0.12 (0.11 – 0.13) 0.004	0.16 (0.13 – 0.18) 0.01
Ovary^(W)^	0.11 (0.07 – 0.14) 0.01	0.08 (0.07 – 0.09) 0.004	0.26 (0.07 – 0.90) 0.26	0.16 (0.13 – 0.18) 0.01
**Caracteres**	***Gibsoniela* Woodland,1935**	**S*errasentis* Van Cleave, 1923**		
				
Body Length	28.32 (17.49 – 46.43) 5.26	15.52 (13.37 – 16.94) 0.67		
N	1 (0.53 – 1.89) 0.23	0.65 (0.19 – 1.50) 0.24		
TN^(L)^	-	0.15 (0.14 – 0.16) 0.004		
Proboscis^(L)^	-	1.44 (1.24 – 1.81) 0.10		
# middle hook rows	-	13		
# rows of hooks on the side	-	16		
Immature proglottids^(L)^	0.14 (0.09 – 0.17) 0.01	-		
Mature proglottids^(L)^	0.15 (0.08 – 0.25) 0.04	-		
Gravid proglottid^(L)^	0.19 (0.08 – 0.43) 0.06	-		
Scolex^(L)^	1.12 (0.89 – 1.33) 0.07	-		
Scolex^(W)^	0.90 (0.75 – 1.01) 0.04			
Suckers	Elongated and triloculated	-		

All measurements are given in mm and are presented in the table as mean, smallest and largest values ​​in parentheses, followed by the standard error.

L: length; W: width; VL: ventricle length; VW: ventricle width; VA: ventricular appendage; N: neck; TN: thorn necklace; A: acetabulum; OS: oral sucker; VS: ventral sucker; AT: anterior testis; PT: posterior testis; RVt: right vitellarium; LVt: left vitellarium.

a Distance anterior to the nerve ring.

## Discussion

The records for endoparasite fauna in *B. surinamensis* are still sparse. In this species, only microparasites in the northern region of Brazil have been described (Monteiro et al., 2019; Araujo et al., 2024). Despite some reports of gastrointestinal parasites in toadfish, there are no records in the literature on the helminth fauna of this fish, which is captured, raised, consumed, and sold in the estuarine region of the Brazilian Amazon. After conducting this study, we were able to observe that the parasitic fauna of *B. surinamensis* is quite diverse, with the phyla Nematoda and Cestoda having high prevalence.

Eiras et al. (2017) recorded *Hysterothylacium reliquens* (Norris & Overstreet, 1975) in Brazil in *B. surinamensis*, in Colombia in *Batrachoides* pacific and *B. boulengeri* and in Guyana in *B. surinamensis*. In our research, *Hysterothylacium* sp. were found in the intestine and *Goezia* sp. in the stomach of hosts from the municipalities of Soure and Bragança, since there is still no record in the literature of the findings reported in the species under study. This suggests that the occurrence of *Hysterothylacium* spp. in *B. surinamensis* is more linked to the species and the morphophysiological characteristics of the parasite-host relationship than to the environment and environmental conditions of the countries where this fish is found.

In this study, we noted the challenge of identifying closely related parasites that have similar morphological characteristics such as Derogenidae and Gorgoderidae. This highlights the need to use molecular tools for accurate identification of these trematodes (Petkevičiūtė et al., 2020). These techniques are relevant to confirm new species of parasites that have not yet been described in the scientific community, as is the case of the helminths described in this research.

Comparing the helminth fauna of the host species studied from Marajó Island and Bragança in Pará is important and shows that the acanthocephalans found in this research were of the genus *Serrasentis* Van Cleave, 1923 and only occurred in fish from Bragança. Rocha et al. (2016) carried out the helminth fauna of *Brachyplatystoma rousseauxii* fish in Amazonian environments, highlighting the importance of studies of parasitic fauna in order to promote greater knowledge of their infestation by parasites in hosts.

*B. surinamensis* is a fish sold mainly in northeastern Pará due to the soft texture of its meat. It is caught by fishing using trawl nets and sold in street markets and in the municipal market in Viseu and Bragança, among other fishing centers (Freire et al., 2011; Monteiro et al., 2019). Even though it is a fish sold in street markets, it still has an unknown economic and social role in Brazil, as reported by Garcia et al. (2017).

Nonetheless, the species *B. surinamensis* is of significant economic importance, as these fish are widely consumed by the riverside population in the study sites. Hence the importance of studying the occurrence of parasites, their prevalence and the parasite-host relationship, thus contributing to the knowledge of the health status of the ichthyological fauna of relevance in fisheries production (Monteiro et al., 2019).

The diversity of helminths found in this research may be related to the fish having low activity during the day, remaining camouflaged on or partially buried in the substrate, and their carnivorous feeding habit, consuming mainly small gastropod mollusks, crustaceans such as crabs and shrimps, as well as small fish, according to Melo et al. (2015) and Mrceniuk et al. (2021), which are part of the life cycle of the helminths recorded here.

## Conclusion

This was the first record of the gastrointestinal helminth fauna of *B. surinamensis* on Marajó Island and in Bragança in the state of Pará, which contributes additional information regarding the Brazilian ichthyofauna. It is worth mentioning that, due to the description of the helminth fauna of *B. surinamensis*, it was possible to observe a high prevalence of parasites belonging to the genus *Gibsoniela* sp., as well as parasites belonging to the genus *Hysterothylacium* sp., as parasites belonging to this group are important agents of zoonoses, however, no helminths were found in the fish muscles. Given the zoonotic potential of anisakid nematodes such as *Hysterothylacium*, their presence in commercially exploited fish species raises important food safety concerns.

## Data Availability

The raw data supporting the results of this study are available upon request from the authors.

## References

[B001] Amato JFR, Amato SB, Von Matter S, Straube FC, Accordi I, Piacentini V, Cândido JF (2010). Ornitologia e conservação: ciência aplicada, técnicas de pesquisa e levantamento..

[B002] Amato JFR, São Clemente SC, Oliveira GA (1990). *Tentacularia coryphaenae* Bosc, 1801 (Eucestoda: *Trypanorhyncha*) in the inspection and technology of the skipjack tuna, *Katsuwonus pelamys* (L.) (Pisces: Scombridae). Atlântica.

[B003] Amato JFR, Boeger WA, Amato SB (1991). Protocolos para laboratório: coleta e processamento de parasitas do pescado..

[B004] Anderson RC, Chabaud AG, Willmott S (2009). Keys to the nematode parasites of vertebrates: archival volume..

[B005] Araujo JP, Székely C, Molnár K, Pereira CMB, Guerreiro SLM, Hamoy IG (2024). Morphology and phylogeny of *Coccomyxa bragantinensis* n. sp. (Cnidaria: Myxozoa) found parasitising the Coco Sea catfish, *Bagre bagre* (Siluriformes: Ariidae), captured off the coast of Northern Brazil. Parasitol Int.

[B006] Bray RA, Gibson DI, Jones A (2008). Keys to the Trematoda..

[B007] Bush AO, Lafferty KD, Lotz JM, Shostak AW (1997). Parasitology meets ecology on its own terms: margolis et al. revisited. J Parasitol.

[B008] Carvalho EL, Santana RLS, Araujo TF, Benigno RNM, Giese EG (2025). Revisiting trematodes in *Cairina moschata* (Linnaeus, 1758) (Anseriformes: Anatidae) in the Brazilian Amazon. Contrib Cienc Soc.

[B009] Eiras JC, Takemoto RM, Pavanelli GC (2010). Diversidade dos parasitas de peixes de água doce do Brasil..

[B010] Eiras JC, Velloso AL, Pereira J (2017). Parasitos de peixes marinhos da América do Sul..

[B011] Eiras JC (1994). Elementos de Ictioparasitologia..

[B012] Espírito Santo RV, Isaac VJ, Silva LMA, Martinelli JM, Higuchi H, Saint-Paul U (2005). Peixes e camarões do litoral bragantino, Pará, Brasil..

[B013] FAPESPA (2023). Estatística municipal – Bragança.

[B014] Freire JL, Silva BB, Souza AS (2011). Aspectos econômicos e higiênico-sanitários da comercialização do pescado no município de Bragança (PA). Biota Amazôn.

[B015] Garcia J, Alves GA, Oliveira JEL, Carvalho AR (2017). First record of *Batrachoides surinamensis* (Bloch & Schneider, 1801) and *Canthidermis maculata* (Bloch, 1786) (Pisces: Teleostei) from Rio Grande do Norte, northeastern coast of Brazil. Check List.

[B016] Gibson DI, Jones A, Bray RA (2002). Keys to the Trematoda..

[B017] Brasil (2024). Cidades e Estados: Soure.

[B018] Khalil LF, Jones A, Bray RA (1994). Keys to the Cestode parasites of vertebrates..

[B019] Léopold M (2004). Poissons de mer de Guyane..

[B020] Luque JL, Pereira FB, Alves PV, Oliva ME, Timi JT (2017). Helminth parasites of South American fishes: current status and characterization as a model for studies of biodiversity. J Helminthol.

[B021] Melo FAG, Dutra EA, Viana JQ, Araújo TM, Souza ASF, Moura IS (2015). Projeto Pesca Solidária: guia de identificação dos peixes do estuário dos rios Timonha e Ubatuba..

[B022] Menezes NA, Buckup PA, Figueiredo JL, Moura RL (2003). Catálogo das espécies de peixes marinhos do Brasil..

[B023] Molento MB, Almeida JCR, Hamann W, Braz FSF, Bier D, Vieira DL (2017). Análise do parasitismo por nematoides da família Anisakidae em peixes marinhos provenientes do litoral paranaense, Brasil. Arch Vet Sci.

[B024] Monteiro EP, Silva DT, Hamoy I, Sanches O, Matos ER (2019). Morphological and molecular characteristics of *Kudoa viseuensis* n. sp. (Myxosporea: Multivalvulida), found in the muscle of *Batrachoides surinamensis* (Teleostei: Batrachoididae) in the Brazilian Amazon region. Acta Protozool.

[B025] Mrceniuk AP, Caires RA, Carvalho-Filho A, Rotundo MM, Santos WCRD, Klautau AGCDM (2021). Peixes teleósteos da costa norte do Brasil..

[B026] Neves E (2020). Participação comunitária na gestão de recursos pesqueiros na Reserva Extrativista Marinha de Soure, Amazônia Marajoara (PA). GeoTextos.

[B027] OTCA (2018). Programa de Ações Estratégicas Estratégia Regional para a Gestão Integrada dos Recursos Hídricos da Bacia Amazônica.

[B028] Oyarzún-Ruiz P, González-Acuña D (2020). Colecta, preparación e identificación de parásitos. Parasitol Latinoam.

[B029] Petkevičiūtė R, Zhokhov AE, Stunžėnas V, Poddubnaya LG, Stanevičiūtė G (2020). *Phyllodistomum kupermani* n. sp. from the European perch, *Perca fluviatilis* L. (Perciformes: Percidae), and redescription of *Phyllodistomum macrocotyle* (Lühe, 1909) with notes on the species diversity and host specificity in the European *Phyllodistomum* spp. (Trematoda: Gorgoderidae). Parasit Vectors.

[B030] Petrochenko VI, Skrjabin KI (1971). Israel program for scientific translations..

[B031] Piorski NM, Nunes JLS (2010). A case of albinism in *Batrachoides surinamensis* (Batrachoidiformes: Batrachoididae) from north-eastern Brazil. Mar Biodivers Rec.

[B032] Rocha CAM, Junior CAMR, Silva IHF, Alcântara ME, Bisneto MQD, Baker PKB (2016). Aspectos ecológicos da helmintofauna de *Brachyplatystoma rousseauxii* (Siluriformes: Pimelodidae) da Baía do Marajó, estado do Pará, Brasil. Vet Zootec.

[B033] Sures B, Nachev M, Selbach C, Marcogliese DJ (2017). Parasite responses to pollution: what we know and where we go in ‘Environmental Parasitology’. Parasit Vectors.

